# The Potential Effect of Intravenous Calcitriol on the Ischemia-Reperfusion Process and Inflammatory Biomarkers in Patients Following Percutaneous Coronary Intervention (PCI)

**DOI:** 10.22037/ijpr.2019.112469.13778

**Published:** 2019

**Authors:** Farzaneh Dastan, Jamshid Salamzadeh, Saeed Alipour-Parsa, Babak Sharif Kashani, Mohammad Mahdi Hashempour

**Affiliations:** a *Clinical Pharmacy Department, School of Pharmacy, Shahid Beheshti University of Medical Sciences, Tehran, Iran. *; b *Chronic Respiratory Disease Research Center, NRTLD, Shahid Beheshti University of Medical Sciences, Tehran, Iran.*; c *Department of Cardiology, Cardiovascular Research Center, Shahid Beheshti University of Medical Sciences, Tehran, Iran. *; d *Tobacco Prevention and Control Research Center, NRTLD, Tehran, Iran.*

## Abstract

This study aimed to investigate the efficacy of calcitriol on Ischemia-reperfusion Injury (IRI) and inflammatory biomarkers in patients with non-ST-segment elevation acute coronary syndromes (NSTEACS) undergoing elective Percutaneous Coronary Intervention (PCI). A total of 72 patients with NSTEACS were randomly divided into two groups: (1) the calcitriol-treated group, treated with three mcg intravenous calcitriol administered before PCI (n = 36) and (2) the control-treated group (n = 36) The serum high-sensitivity C-reactive protein (hs-CRP), high-sensitivity interleukin-6 (hs-IL-6), creatinine kinase (CK)-MB and cardiac troponin I (cTnI) levels were measured before PCI and 24 h after PCI in both groups. The patients were followed up for the detection of the prevalence of major adverse cardiac events (MACE) in 180 days after PCI in both groups. Compared to pre-PCI, the serum hs-CRP, hs-IL-6, CK-MB, and cTnI levels were increased at 24 h after PCI (all *p *< 0.05) in both groups. However, change in the levels of hs-CRP and hs-IL-6 were significant (*p *= 0.04 and *p *= 0.02, respectively). Changes in the levels of CK-MB and cTnI were non-significant (*p* = 0.15 and *p *= 0.39, respectively). No MACE (death, Q wave MI, target vessel revascularization, ischemic stroke) was detected in any patient in any group during a 3-month follow-up. Administration of calcitriol in patients with non-ST-segment elevation acute coronary syndromes undergoing elective PCI can attenuate the increase in serum inflammatory biomarkers in the serum (hs-CRP and hs-IL-6) and thus decrease the inflammatory reaction caused by PCI.

## Introduction

Cardiovascular disease (CVD) is one of the major causes of mortality and morbidity in the world (1). Among CVDs, acute coronary syndrome (ACS) is associated with significant complications (2, 3). Coronary artery atherosclerosis is known as a chronic inflammatory process that may lead to ACS (4, 5).

Currently, the reperfusion strategy is the standard treatment for acute myocardial ischemia (AMI). This strategy contradictory may cause cardiomyocytes dysfunction and worsens tissue damage (reperfusion injury) (5) Ischemia-reperfusion (IR) injury induces an inflammatory response that causes multi-organ dysfunction. IR injury can result in about 30% to 40% mortality in the intensive care unit (6).

Percutaneous Coronary Intervention (PCI) may cause damage to the vessel wall, leading to localized inflammation of the coronary artery and vascular endothelium damage (2). and inflammatory response (7).

Inflammatory biomarkers are used to evaluate the prognosis of coronary heart disease associated with inflammation, including left ventricular dysfunction, AMI, or PCI-operated patients (8).

Traditionally, vitamin D has been recognized as the main regulator of calcium and phosphorus homeostasis, as well as bone metabolism (9). In the Intermountain Healthcare System study, which was conducted on more than 41,000 people, the association between vitamin D deficiency and coronary heart disease (CHD), MI, heart failure, stroke, hypertension and type 2 diabetes was proven (10). In recent studies, it has been shown that calcitriol suppresses the production of pro-inflammatory mediators in adipocytes, preadipocytes, monocytes, and macrophages (10).

Based on the pleiotropic effects of calcitriol on CHD and inflammation, this study has been designed to evaluate calcitriol effects on IR injury and inflammation in patients undergoing PCI.

*Method*

*Ethics*

This study was approved by the Ethics Committee of Shahid Beheshti University of Medical Sciences and was registered in the Iranian Registry of Clinical Trials (ID: IRCT20151227025726N10). All patients provided written informed consent.

*Study Design and Setting*

This is a prospective randomized, single-blind, clinical trial that was conducted in a referral hospital for cardiovascular diseases in Tehran from October 2017 to September 2018.

The sample size calculation was based on the power of 80% and the level of confidence of 95%; the level of significance (α) was assumed to be 0.05. We selected the highest number for the sample size. 

*Patients*

All patients who were more than 18 years old, referring to Shahid Modarress cath lab for elective PCI with stent placement enrolled in the study. The exclusion criteria were patients with: (1). acute ST-segment elevated MI (STEMI); (2). recent history (within 6 months) of MI; (3). coronary artery bypass grafting (CABG); (4) recent vitamin D supplementation (within 1 month before PCI); (5). unsuccessful PCI; (6). hypercalcemia; (7). active metabolic bone disease; (8). renal or hepatic dysfunction; (9). left ventricular ejection fraction less than 30%; (10). contraindications or hypersensitivity to calcitriol; (11). recent (1 week) consuming anti-inflammatory drugs (except aspirin and statin); (12). breastfeeding or pregnant women; (13). the inability to fill out or understand the consent form.

Patients’ demographic data, including sex, age, weight, height, and body mass index (BMI), were recorded. Also, drug and medical history, laboratory data, and positive family history of cardiovascular disease were documented.

All patients were randomized to the calcitriol-treated group or the control group by the systematic randomization method using computer-generated random numbers. All the patients in the intervention group were received three mcg intravenous (IV) bolus dose of calcitriol (3 ampules, CALCITRIOL ORPHATEB 1 mcg/mL injection, Mefar co., Turkey) 2 h before PCI. 

All study participants received the standard PCI pre-treatment protocol of clopidogrel 300-600 mg, aspirin 300 mg, and intravenous heparin with a target activated clotting time of 250–300 seconds. All patients received 100 ± 25 ml of the contrast agent visipaque (iodixanol) during PCI. All PCIs were done by one interventional cardiologist according to the standard practice guidelines. The practitioner and the laboratory staff were blinded to the allocation. All patients were followed up for 3 months for the major adverse cardiovascular events (MACE) including death, Q-wave MI, target vessel revascularization, and ischemic stroke.

The creatinine kinase-MB (CK-MB), cardiac troponin I (cTnI), high-sensitivity C-reactive protein (hs-CRP) and high-sensitivity Interleukin-6 (hs-IL-6) levels were measured at the baseline (before giving calcitriol) and 24 h after PCI. Venous blood samples were obtained in tubes, EDTA for hs-CRP and citrate for IL-6. The tubes were centrifuged within 30 min of sample collection at 2000 rpm for 10 min at room temperature and frozen at -70 °C until analysis. Plasma concentration of CK-MB and cTnI were measured using spectrophotometry (Spectrophotometer UH4100, Hitachi, Japan) chemiluminescent immunoassays technique (IMMULITE 2000, Siemens, Germany), respectively. Plasma concentrations of hs-CRP and hs-IL-6 were analyzed using an ELISA technique (Stat Fax 2100, GMI, USA). The minimal detectable concentration of CK-MB, cTnI, hs-CRP and hs-IL-6 in blood were 1 U/L, 0.02 ng/mL, 0.02 mcg/mL and 0.03 pg/mL, respectively.

The primary outcome was the comparison of CK-MB and cTnI levels at the baseline and 24 h after PCI for assessing peri-procedural myocardial injury (PMI) as well as a comparison of hs-CRP and hs-IL-6 before and 24 h after PCI to assess the anti-inflammatory effect of IV calcitriol. The secondary outcome was the incidence of MACE (death, Q wave MI, target vessel revascularization, ischemic stroke) during a 3-month follow-up period considering the incidence timeline of MACE and the limitation in the study duration.

Statistical analysis was performed using statistical package for social sciences software (SPSS version 20.0). The normality distribution of data was assessed by the Kolmogorov–Smirnov test. Mann-Whitney and independent-sample *t*-test were used to compare means between the different groups. Chi-square and Fisher’s exact test were applied to perform the frequency analysis. Continuous data were shown as mean ± standard deviation (SD). *p*-value < 0.05 was assumed as statistically significant.

## Results

*Baseline and procedural characteristics*

Clinical and procedural features in the calcitriol-treated (36 patients) and control (36 patients) groups are shown in Table 1. There were no significant differences between the two groups in age, gender, BMI, the prevalence of cardiovascular risk factors (smoking, hyperlipidemia, family history, hypertension, diabetes mellitus, and MI), left ventricular function, serum creatinine, estimated glomerular filtration rate, and baseline vitamin D blood level. Angiographic and procedural characteristics were also comparable in both groups. The lesion number (*p* = 0.03) and surgery duration (*p* = 0.03) were significantly higher in the treatment group comparing to the control group (Table 2). Drug-eluting stents were used in all patients. Procedural success was obtained in all patients, and there were no procedure-related complications in either group.

*Inflammatory and Cardiac biomarkers*

Distribution of the baseline CK-MB, cTnI, hs-CRP, and hs-IL-6 were non-normal and had no significant differences except cTnI. (Table 2). All the mentioned biomarkers increased significantly 24 h after PCI compared to the baseline values except CK-MB. (Table 3)

The distribution of change in levels of cTnI, hs-CRP, and hs-IL-6 was non-normal, except CK-MB between the two groups. The results revealed that the changes in the level of hs-CRP (95% confidence interval (95% CI): 0.1 to 2.56, *p* = 0.04) and hs-IL-6 (95% CI: 0.29 to 3.65, *p* = 0.02) were significant. The change in the levels of CK-MB was not significant between the two groups (95% CI: -4.20 to -0.67, *p* = 0.15). The results also showed that the change in the levels of cTnI was not statistically significant (median 0.02 [interquartile range (IQR): 0 to 0.05]; median 0.06 [IQR: -0.01 to 0.19]) in control and the treatment groups, respectively (*p *= 0.39). (Table 4).

*Major adverse cardiac events*

The incidence of MACE (death, Q wave MI, target vessel revascularization, ischemic stroke) during a 3-month follow-up evaluated, and no MACE was detected in any patient in any group.

## Discussion

The results of the current study showed the significant effect of calcitriol on modulating the inflammatory biomarkers.

The American College of Cardiology/American Heart Association (ACC/AHA) recommends the measurement of post-PCI biomarkers to determine PMI and evaluate the outcome of treatment (4, 11, 12) The European Society of Cardiology (ESC) recommends measuring the hs-CRP level as a part of the risk assessment program for patients with the risk of cardiovascular disease (class II b) (1, 13). In the last decade, systemic inflammatory biomarkers have been well studied, and some have been associated with death, myocardial infarction, or stent restenosis after PCI (14, 15).

 In the current study, hs-CRP and hs-IL-6 were significantly lower in the calcitriol group comparing to the control group after PCI. The results were similar to the recent studies, which showed the anti-inflammatory effect of vitamin D and calcitriol (10, 11). In another study, the effect of atorvastatin on lowering CRP was reported. (16). Among inflammatory biomarkers, the CRP is widely used to evaluate the inflammatory conditions. Hs-CRP is mentioned as a biomarker of inflammation in the studies. Higher levels of hs-CRP before and after PCI are associated with a higher risk of adverse cardiac events and MACE in recent studies (3, 7, 14, 17-21). IL-6 is an upstream inflammatory biomarker that acts as a mediator for intensifying inflammatory response. IL-6 also plays an essential role in initiating and developing the process of atherosclerosis. The level of IL-6 is relevant to the incidence and prognosis of cardiovascular disease, which supports the hypothesis of inflammation. The strong association between IL-6 and the risk of developing MACE is proven in recent studies (3, 20-22).

Although Baseline cTnI, surgery duration and lesion number were significantly higher in the treatment group, hs-CRP and IL-6 were reduced significantly in the mentioned group comparing to the control group. These changes showed the beneficial effect of calcitriol on inflammation in patients during PCI.

There were no significant differences in CK-MB and cTnI levels between the two groups in our study. The results were similar to the recent studies, which showed vitamin D, and carnitine administration has no significant effect on cardiac biomarkers (11, 23).

Compared to CK-MB and myoglobin, cardiac troponins are more sensitive and accurate factors to show damage to cardiomyocytes (1). The increase in CK-MB levels, as well as cardiac troponin, are associated with short-term and long-term adverse events after PCI (24).

No MACE reported in two groups of this study. We decided to follow up on the patients for three months based on the MACE incidence timeline in recent studies (25). and also the study duration limitation. The MACE results in our study may associate with the low sample size or the study design. In general, PMI occurs due to the embolization of plaque thrombosis, platelet aggregation, clot formation, coronary artery spasm, oxidative stress, and inflammation during the PCI procedure (11, 15, 16).

Myocardial IR injury is a complex process that involves many interacting factors, including reduced levels of ATP, hydrogen ion accumulation, calcium accumulation, and an increase in the production of active oxygen components in the cell. All of these factors together cause cell damage and subsequent death of myocyte cells (6, 26). The oxidative stress results in the accumulation of oxidants (due to an increase in the production of reactive oxygen species) or reduced ability to scavenge them, which ultimately results in cellular damage to cardiomyocytes (5). 

According to new findings, low levels of vitamin D are associated with an increased risk of cardiovascular disease and mortality. The interference of vitamin D with the pathogenesis of cardiovascular disease has been addressed through its inflammatory and antithrombotic effects. It also inhibits the renin-angiotensin-aldosterone system (RAAS), reduces vascular calcification, and controls the progression of atherosclerosis (11).

Our study was the first randomized clinical trial that evaluated the effects of calcitriol on myocardial injury and ischemia-reperfusion injury in patients undergoing elective PCI. Lesion number and surgery duration were significantly higher in the treatment group comparing to the control group, which confirmed the potential role of calcitriol in attenuating PMI.

As inflammation is one of the most important causes of PMI, calcitriol was selected based on data regarding its anti-inflammatory effects (9).

Considering the limitations of our study, the authors recommend further studies with higher sample size, different or multiple doses of calcitriol, serial measurement of inflammatory biomarkers, and a more extended follow-up period.

**Figure 1:**
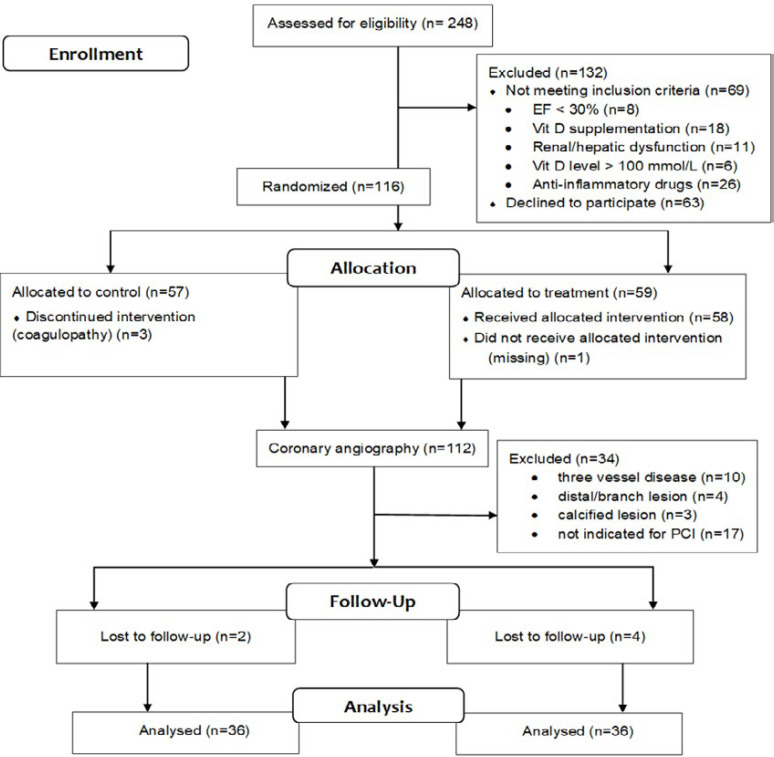
Consort flowchart of the study

**Table 1:** Baseline clinical characteristics of patients

**Characteristics**	**Control (n = 36)**	**Treatment (n = 36)**	**P value**
Age, years	62.5 ± 10.7	63.6 ± 10.4	0.65
Sex (n) Male/female	28/8	27/9	1.0
BMI (kg/m2)	26.7 ± 3.9	28.4 ± 4.8	0.06
Creatinine (mg/dL)	1.23 ± 0.23	1.23 ± 0.22	0.98
eGFR (mL/min)	54.2 ± 19.2	52.4 ± 15.9	0.66
Vitamin D level (nmol/L)	28.43 ± 2.68	23.50 ± 2.45	0.16
Hypertension, n (%)	21 (58.3)	16 (44.4)	0.24
Diabetes mellitus, n (%)	12 (33.3)	8 (22.2)	0.29
Dyslipidemia, n (%)	11 (30.6)	5 (13.9)	0.09
Smoking, n (%)	12 (33.3)	19 (52.7)	0.09
Family history of CAD, n (%)	10 (27.8)	4 (11.1)	0.07
Medications Aspirin Statins ACEI/ARB CCB Nitrates Beta blockers	19 (52.8) 26 (72.2) 23 (63.9) 5 (13.9) 19 (52.8) 18 (50)	22 (61.1) 28 (77.8) 19 (52.7) 3 (8.3) 22 (61.1) 22 (61.1)	
NSTEMI, n (%)	11 (30.6)	9 (25)	0.60
LVEF (%)	47.2 ± 7.9	49.4 ± 7.1	0.21

**Table 2:** Angiographic characteristics and ذaseline biomarkers of the patients

**Characteristics**	**Control (n = 36)**	**Treatment (n = 36)**	***P*** ** value**
Baseline CK-MB (unit/L)	14.3 ± 6.8	16.1 ± 7.6	0.33
Baseline cTnI (ng/mL)	0.18 ± 0.5	0.52 ± 0.9	0.01
Baseline hs-CRP (mg/L)	4.27 ± 4.3	5.75 ± 5.3	0.45
Baseline hs- IL-6 (pg/mL)	3.31 ± 4.6	2.34 ± 1.9	0.47
Surgery duration (min)	104.7 ± 46	121.7 ± 38	0.03
Lesion number (n)	1.4 ± 0.7	1.7 ± 0.7	0.03
Stent number (n)	1.3 ± 0.5	1.6 ± 0.7	0.27

**Table 3:** Inflammatory and cardiac biomarkers (pre and post PCI).

	**Control**			**treatment**		
	pre	post	*P* value	pre	post	P value
CK-MB (unit/L)	14.30 ± 6.83	14.52 ± 6.01	0.81	16.08 ± 7.56	17.81 ± 8.81	0.22
cTnI (ng/mL)	0.18 ± 0.52	0.35 ± 0.79	0.002	0.52 ± 0.81	0.79 ± 1.05	0.03
hs-CRP (mg/L)	4.27 ± 4.34	7.17 ± 4.46	0.001	4.75 ± 5.27	7.65 ± 4.96	< 0.001
hs- IL-6 (pg/mL)	3.31 ± 4.60	6.28 ± 6.03	< 0.001	2.34 ± 1.95	3.61 ± 1.99	< 0.001

**Table 4:** Comparing mean difference changes in the outcome variables

**Characteristics**	**Control (n = 36)**	**Treatment (n = 36)**	***P*** ** value**
CK-MB (unit/L)	0.23 ± 4.69	1.53 ± 5.29	0.15
cTnI (ng/mL)	0.17 ± 0.60	0.26 ± 0.94	0.39
hs-CRP (mg/L)	2.67 ± 2.91	1.33 ± 2.22	0.04
hs- IL-6 (pg/mL)	2.80 ± 4.67	0.84 ± 1.27	0.02

## Conclusion

Calcitriol, as an active form of vitamin D, may reduce the inflammatory biomarkers following PCI. It could have a crucial role in reducing ischemia-reperfusion injury after PCI surgery.
